# Guideline Concordance of Antibiotic Recommendations From Large Language Models in Dentistry: A Vignette-Based Evaluation in Georgian and English

**DOI:** 10.7759/cureus.117183

**Published:** 2026-09-29

**Authors:** Zurab Alkhanishvili, Ketevan Gogilashvili, Nino Gvasalia, Sopio Samkharadze, Landa Lursmanashvili, Luka Nachkebia, Aleksandre Chkhikvishvili, Ali Qasim Tahir, Nana Tsitskishvili

**Affiliations:** 1 Faculty of Medicine, Ivane Javakhishvili Tbilisi State University, Tbilisi, GEO; 2 Faculty of Dentistry, European University, Tbilisi, GEO; 3 Department of Periodontology, Albius Dental Center, Tbilisi, GEO; 4 Department of Public Health, Georgian Association for Public Health and Well-Being, Tbilisi, GEO; 5 Department of Dentistry, International Medical School, Alte University, Tbilisi, GEO

**Keywords:** antimicrobial stewardship, artificial intelligence, dental prescribing, georgia, large language models, low-resource languages, odontogenic infection

## Abstract

Introduction: Inappropriate antibiotic prescribing in dentistry is a recognised stewardship concern. Large language models (LLMs) are readily accessible sources of prescribing information, but dental evaluations have been conducted almost entirely in English. We compared the guideline concordance of matched Georgian and English LLM recommendations for common dental presentations in a setting in which we could identify no national dental antibiotic prescribing guideline.

Materials and methods: In this preregistered vignette study, 20 standardised scenarios (10 antibiotic-indicated, 10 not) were submitted in Georgian and English to the free-tier interfaces of Claude (Anthropic, San Francisco, USA), ChatGPT (OpenAI, San Francisco, USA) and Gemini (Google LLC, Mountain View, USA) on three occasions (360 responses). Responses were normalised, blinded to system and run, and coded by two dentists against a key based on Scottish Dental Clinical Effectiveness Programme (SDCEP), American Dental Association (ADA) 2019 and American Heart Association (AHA) 2021 guidance on five domains: decision to prescribe, agent, dose and frequency, duration, and referral advice. Disagreements were adjudicated and the regimen domains were re-scored under clarified rules. The primary outcome was full concordance on all applicable domains in the first run, compared across 60 matched scenario-system pairs with the exact McNemar test.

Results: Full concordance was 57/60 (95.0%, 95% CI 86.3 to 98.3) for English and 46/60 (76.7%, 64.6 to 85.6) for Georgian prompts, a paired difference of 18.3 percentage points (95% CI 7.5 to 29.2; p = 0.003); 12 pairs were concordant in English only and one in Georgian only. The decision to prescribe was concordant in all 360 responses and referral advice in 59/60 per language. In antibiotic-indicated scenarios, an accepted first-choice agent was given in 30/30 English and 21/30 Georgian responses (p = 0.004); dose and duration concordance was also lower in Georgian (16/27 and 15/27 versus 26/27 and 25/27) but was scored discordant whenever the agent was discordant. All 30 no-antibiotic pairs were concordant in both languages. The between-system comparison was not significant (Cochran's Q p = 0.14). The agent code was reproducible across three runs in 29/30 English and 26/30 Georgian antibiotic-indicated cells (p = 0.25). No first-run response met a prespecified safety-critical error criterion (0/60 per language, 95% CI 0.0 to 6.0).

Conclusions: Decision concordance was preserved across languages, but full concordance with a UK stewardship standard was lower for Georgian prompts, chiefly because the first-choice agent was broader than the reference accepts. LLM-generated regimen details should be checked against an authoritative source. The findings underscore the value of accessible, authoritative Georgian-language dental prescribing guidance.

## Introduction

Antimicrobial resistance remains a major global health challenge. In 2021, an estimated 1.14 million deaths were attributable to bacterial antimicrobial resistance and 4.71 million were associated with it, and without substantial improvement in prevention, access to effective treatment and antimicrobial stewardship, attributable deaths are projected to reach approximately 1.91 million a year by 2050 [1]. Dentistry is an important target for stewardship. Dentists account for approximately 10 per cent of antibiotic prescribing worldwide, and a large proportion of dental antibiotic prescriptions are inappropriate [2].

For common acute dental conditions, guidance emphasises local treatment over systemic antibiotics. The American Dental Association (ADA) recommends against antibiotics for most pulpal and periapical conditions, reserving them for systemic involvement or a substantial risk of progression [3], and Scottish Dental Clinical Effectiveness Programme (SDCEP) advises antibiotics only when local measures fail or when cellulitis, spreading infection or systemic involvement is present [4]. Appropriate prescribing therefore depends on the decision to prescribe and, where treatment is warranted, on the agent, dose, frequency and duration.

In Georgia, a nationally representative survey of 1,876 adults found that only 32 per cent knew that antibiotics are ineffective against viral infections [5], and a qualitative study of dentists identified patient expectations, defensive prescribing, diagnostic uncertainty and gaps in guideline adherence as drivers of prescribing [6]. We could identify no Georgian national guideline on antibiotic prescribing in dentistry in the Ministry of Health's approved-guideline register, the professional associations or the Georgian-language literature, so a clinician seeking an authoritative answer must look outside the formal system, and readily accessible decision support is of particular relevance to stewardship.

General-purpose large language models (LLMs) answer clinical questions immediately and are therefore an easily accessible source of health information, but evidence on their reliability for dental antibiotic decisions is limited. A 2026 evaluation of ChatGPT and Gemini found high scores on general questions about dental antibiotic use but limitations in patient-specific recommendations [7]; four conversational systems generally identified antibiotic indications in endodontic scenarios, with frequent incomplete answers [8]; and repeated testing on endodontic prophylaxis showed variable accuracy and repeatability [9]. In general practice vignettes that included dental infections, six LLMs identified the decision to prescribe and an appropriate agent in most cases but gave a guideline-concordant dose and duration in far fewer and were less reliable with country-specific guidance [10]; a comparison of 14 LLMs found substantial between-system differences in agent, dose and duration [11]. A model may thus reach a broadly appropriate decision while giving an imperfect regimen.

Whether performance is preserved across languages is a further open question. Health answers were less correct, consistent and verifiable in Spanish, Chinese and Hindi than in English [12], multilingual medical benchmarks show gaps between English and other languages [13], and accuracy on medical questions varies with language even among German, French and Italian [14]. Georgian is a low-resource Kartvelian language with its own script; LLM reasoning in Georgian has only begun to be benchmarked and clinical prescribing has not been addressed [15]. To our knowledge, no study has tested whether the same dental scenarios yield equally guideline-concordant recommendations in Georgian and in English.

We therefore conducted a preregistered, vignette-based evaluation of three general-purpose consumer LLM interfaces to compare the guideline concordance of antibiotic recommendations generated from matched Georgian- and English-language prompts. The primary objective was to determine whether full guideline concordance differed by language, using an explicit reference standard covering the decision to prescribe, the antibiotic agent, dose and frequency, duration, and referral advice. Secondary objectives were to compare performance across systems, to assess the reproducibility of recommendations across repeated runs, and to identify predefined potentially harmful errors [16]. We hypothesised that full concordance would be lower for Georgian than for English prompts (H1), that concordance would vary across domains and be lowest for dose and duration (H2), that responses would not be fully reproducible across runs, with lower reproducibility in Georgian (H3), and, as an exploratory question, that concordance would differ between systems (H4).

## Materials and methods

Design and registration

This was a cross-sectional, vignette-based evaluation of the antibiotic recommendations returned by three consumer LLM chat interfaces to matched Georgian and English prompts, preregistered on the Open Science Framework (OSF) before data collection (7 September 2026) [16]. Twenty scenarios were submitted to each of three systems in each language on three occasions, giving 360 responses; the primary analysis used the 120 first-run responses and the repeat runs served the reproducibility analysis. The study analysed only model-generated text in response to fictional vignettes and involved no patients or human participants. The experts who validated the scenarios and the dentists who pre-tested the Georgian prompts acted as consultants on the study instruments: their ratings and comments were used to revise the materials and are reported in aggregate, no personal or outcome data about them were collected, and they were not the object of study. The Biomedical Research Ethics Committee of the Georgian Dental Association reviewed the protocol and determined that the study involves no human subjects and is exempt from full ethical review (certificate GSA-BREC-2026-014, 13 August 2026); informed consent was not applicable. The principal investigator is vice president of the Georgian Dental Association and other authors are members of it; none took part in the committee's deliberation or decision, and the committee had no role in the design, conduct, analysis or reporting of the study. Departures from the registered protocol are recorded in a deviations log deposited with the project (items cited below as D1 to D22).

Clinical scenarios and prompts

Twenty standardised vignettes were purposively written to cover common situations in which a general dentist decides whether to prescribe an antibiotic; they are a benchmark set, not a frequency-based sample of practice. Ten describe conditions in which an antibiotic is not indicated by the reference guidelines (S01 to S10: irreversible pulpitis, symptomatic apical periodontitis, localised abscess with drainage available, alveolar osteitis, prophylaxis request before routine extraction, localised pericoronitis, periodontal abscess without systemic involvement, chronic abscess with sinus tract, extraction outside an endocarditis prophylaxis category, post-endodontic pain) and ten in which it is (S11 to S20: spreading odontogenic infection, apical abscess with systemic involvement, pericoronitis with systemic involvement, infection with airway compromise, impaired host defence, severe spreading infection in penicillin allergy, necrotising gingivitis, post-extraction infection, endocarditis prophylaxis in an indicated patient, severe infection in a young child). Two scenarios sit where the sources diverge (S16, agent in penicillin allergy; S19, prophylactic dose) and the key accepts either source there.

Every scenario was submitted with the same fixed wording. The English frame read: "I am a general dentist. A patient presents with the following. [vignette] Should this patient be prescribed an antibiotic? If an antibiotic is indicated, which antibiotic, at what dose and frequency, and for how long? Are there any signs that would require urgent referral or close monitoring?" The Georgian prompts used the same frame in translation, produced as described below; all 40 prompts as sent are deposited with the project (Prompts as sent 2026-09-09). Content validity of the English scenarios and key was assessed before translation by seven dental or medical experts, who rated each scenario for relevance and clarity on a four-point scale with comments; the item content validity index (proportion rating 3 or 4) was computed per scenario and criterion against thresholds fixed in advance (0.78 per item, 0.90 scale average) and was 1.00 for all 20 scenarios on both criteria (one expert left one scenario unrated). On the comments, radiographic findings were added to nine scenarios and wording clarified in three; no reference answer changed. The vignettes were translated into Georgian by a bilingual medical translator and back-translated by a second translator who had not seen the English source; the investigator compared the two English versions, and the discrepancies found (a tooth number, a lost parenthesis, severe rendered as acute in three scenarios) were corrected and re-checked. No machine translation or LLM was used at any stage, which both translators confirmed in writing. The Georgian prompts were then pre-tested by seven practising dentists (5 to 24 years in practice, independent of the translation and of the team), who answered for each scenario whether it was comprehensible and whether it read naturally, with a suggested wording for any no, and were asked not to judge the clinical content. Of 46 comments, a change was made only where at least two dentists raised the same point and one supplied a replacement: ten terminology edits across twelve scenarios, meaning unchanged, after which the prompts were frozen on 9 September 2026. Four vignettes outside the study set were run on all three systems in both languages as a pilot to test the procedure and calibrate the coding manual with the two raters originally appointed; pilot responses were not analysed. The translation pack, back-translation audit, validation ratings and pre-testing log are deposited with the registration. Prompts were single turns with no system instruction, attachment or prior context.

Reference standard

Responses were scored against a scenario-by-scenario reference key. Agent, dose and duration were taken from the SDCEP Drug Prescribing for Dentistry guidance, accessed on 4 September 2026 (bacterial infections section updated May 2026, with a later clarification on review timing) [4]; the decision to prescribe was scored against the ADA 2019 guideline where the scenario fell within its scope [3] and against SDCEP elsewhere; endocarditis prophylaxis against the AHA 2021 statement with its published corrections [17] and the SDCEP prophylaxis advice [18]. SDCEP was the primary standard because it gives agent, dose, frequency and duration for the full range of presentations used, including periodontal conditions, prophylaxis and paediatric dosing, whereas ADA 2019 covers pulpal and periapical conditions in immunocompetent adults only.

The key was frozen as version 7 before collection and amended twice after re-reading the SDCEP source: version 7.1 (11 September 2026, before coding) widened the accepted duration for S13 and S17 from 3 to 3 to 5 days, and version 7.2 (13 September, after coding) returned it to 3 days because the source did not support the widening. After the first coding round, a review of the adjudicated disagreements showed that two interpretive rules had been applied inconsistently, and they were written into an addendum to the coding manual: a stated range is concordant if it contains the accepted value, and where more than one regimen is offered the one presented as first choice is scored (options of equal standing are concordant if either is accepted). Domains 2 to 4 of the 60 first-run antibiotic-indicated responses were then re-scored by both raters against version 7.2 and the addendum, independently and blind to system, run and previous codes; they agreed on 160 of 168 applicable cells, and the eight differing cells were adjudicated by the principal investigator, blind to system and run but not to language, with the three clarifications this required (overlapping ranges, dose and duration under a discordant agent, combination regimens) added to the addendum before analysis. Second- and third-run agent codes were then re-scored under the same rule by both raters, with agreement on all 120 cells. All figures below use this dataset; earlier datasets and their effects are retained in the deposit and documented in the deviations log. Raters coded against the key alone and recorded any disagreement with it in a note.

Systems and data collection

Three consumer chat interfaces were evaluated: Claude (claude.ai, Anthropic, San Francisco, USA), ChatGPT (chatgpt.com, OpenAI, San Francisco, USA) and Gemini (gemini.google.com, Google LLC, Mountain View, USA), each on one free account created for the study, to evaluate what a clinician receives by default. The protocol had also specified paid subscriptions to guard against fallback to lighter models; the two requirements could not both be met, because a paid plan does not expose the free-tier default model, and the free-tier requirement was retained (D1). Eighty responses collected on paid tiers before this was resolved are deposited as exploratory material and not analysed (D2).

The configuration was verified from each interface on 10 September 2026 with screenshots (Table 1). Claude and Gemini display the model name; the ChatGPT free plan does not, and its self-report (GPT-5.6 Luna) is recorded as weak evidence. Memory, personalisation and custom instructions were disabled through temporary or incognito modes. Web search was switched off in Claude and ChatGPT; Gemini provides no way to disable it, so all Gemini responses were generated with web search available.

**Table 1:** System configuration for the registered dataset Claude: Anthropic, San Francisco, USA; ChatGPT: OpenAI, San Francisco, USA; Gemini: Google LLC, Mountain View, USA

Label	System	Plan	Model as displayed	Chat mode	Web search	Other settings
Model_A	Claude (claude.ai)	Free	Sonnet 5	Incognito	Off	Effort Medium
Model_B	ChatGPT (chatgpt.com)	Free	Not displayed (self-reported GPT-5.6 Luna)	Temporary	Off	Unpersonalised; Think mode not used
Model_C	Gemini (gemini.google.com)	Free	3.6 Flash	Temporary	On (cannot be disabled)	None

Responses were collected on 10 and 11 September 2026, within the protocol window, each prompt in a fresh session. The registered randomised run order was not followed: responses were collected in scenario order, the three English runs before the three Georgian runs within each scenario, one system at a time (D4). No usage-limit switch to another model occurred. The protocol's pre-specified reply to a clarifying question was never triggered (D7). Three responses were recollected after capture failure, not content (manifests, deviations log).

Normalisation and blinding

Before rating, every response was converted to uniform plain text: self-identification and characteristic formatting were removed, markers and spacing normalised, and 19 Gemini responses had mathematical notation converted to ordinary characters; no clinical wording was altered (D5). Blinded identifiers B001 to B360 were assigned in a single random shuffle after collection was complete (D6). Rater packets showed the identifier, the scenario number and the prompt language, which cannot be concealed; they were checked mechanically for system names, model labels and run numbers.

Outcome measures

Each response was coded on five domains against the key for its scenario: d1, the decision whether an antibiotic is indicated; d2, the agent; d3, dose and frequency; d4, duration; d5, referral and red-flag advice; each 1 (concordant) or 0 (discordant), with 9 for d1 when no decision was given and 2 for d3 or d4 when an agent was named without a dose or duration (treated as discordant). A stated range was concordant if it contained the accepted value or, where the accepted value is a range, if the two overlapped (addendum A2, A2a). Where more than one regimen was offered, the one presented as first choice was scored; options of equal standing were concordant if either was accepted (A3, A3a). When the first-choice agent was discordant, dose and duration were not assessed and were coded 0 (A2b). In the 10 no-antibiotic scenarios, d2 to d4 were not applicable; in S14 (airway compromise), any plausible agent is accepted provided emergency transfer is stated, and dose and duration were not scored, so dose and duration were assessable in nine scenarios and the agent in ten. The primary outcome, composite full concordance, is concordance on every applicable domain. A separate variable recorded whether the response met any criterion on a closed list of safety-critical errors defined a priori: prescribing where clearly not indicated, a beta-lactam despite documented penicillin anaphylaxis, failure to state emergency transfer with airway compromise, a multi-day course for single-dose prophylaxis, exceeding the paediatric dose ceiling, and withholding antibiotics despite spreading infection.

Seven descriptive variables were coded (delayed prescription suggested, clarifying question, refusal, antibiotic not mentioned, language mismatch, source cited, deferral to local guidance). The coding manual, version 4, was frozen on 9 September 2026. First-run responses were coded on all variables; second- and third-run responses on d1 and d2 only. Three coding rules bear on the results. A delayed or back-up prescription, that is, advice not to start an antibiotic at the visit but to obtain one if the condition worsened, was scored as a concordant decision in the no-antibiotic scenarios, because no antibiotic was recommended at the visit, and was recorded separately as a descriptive variable. A combination regimen was scored as a whole against the key, so that adding an agent the key does not specify to an accepted agent was discordant. For the paediatric scenario (S20, 14 kg) the accepted dose bands were amoxicillin 250 to 420 mg three times daily or phenoxymethylpenicillin 125 to 175 mg four times daily, from the SDCEP paediatric tables; an adult dose was discordant.

Raters, agreement and adjudication

The responses were coded independently by two authors (N.G. and A.Q.T., R1 and R2), dentists fluent in both languages who had not taken part in scenario development. They replaced the two raters who had completed the calibration rounds on the pilot set and then became unavailable; the replacements did not repeat the calibration and coded from the frozen manual, the key and the packet (deviations log). Both were assigned all 120 first-run responses on all variables; the 240 repeat-run responses were divided between them by stratified random allocation with a 20 per cent duplicate subsample of 48 coded by both, so that 168 responses were double-coded. Raters did not discuss any response until both had finished. Agreement was calculated on the two independent files as percentage agreement and Cohen's kappa with bootstrap 95 per cent confidence intervals on the cells where the domain was applicable and a code was required; a rater's NA entered where a code was required was kept as a category.

Twenty-nine domain-level disagreements arose on 23 responses; the raters resolved none by discussion, and all were adjudicated by the principal investigator (Z.A.), who saw the blinded response, the key and the two codes without rater identity, system or run. The adjudicator agreed with R2 in 24 of 29, including all eight English-language disagreements. Eight of the 29 were NA entries by R1 in the agent domain of S14 rather than opposing codes; the adjudicator recorded 1, and six further such entries on single-rater repeat-run S14 responses were set to 1 by the investigator after verification. R1 left the descriptive variables blank, so those results rest on R2's codes. The dataset records the origin of every consensus code.

Use of artificial intelligence tools

An LLM assistant (Claude, Anthropic) was used under the direction of the principal investigator to prepare study documents, generate the scripts that normalised, blinded and allocated the responses, compute statistics, check file consistency and draft manuscript text, all verified and edited by the authors. The assistant did not generate, code, rate or adjudicate any response, did not translate any prompt, and made no coding or analytic decision. It is a product of one of the three vendors evaluated; it was accessed through a separate paid interface, was not the free-tier system under test, and had no part in producing the evaluated responses. The deposited analysis script was checked by the authors and reproduces every figure in this article from the deposited data.

Statistical analysis

The primary and secondary analyses were prespecified; the registered plan named Microsoft Excel, and the analyses were run in Python (3.12.3, SciPy 1.17.1) to compute exact tests directly (D15), by a single deposited script. Proportions carry Wilson 95 per cent confidence intervals. The primary hypothesis (H1), lower composite concordance for Georgian prompts, was tested on the 60 first-run scenario-system pairs with the exact McNemar test, the paired difference with a Wald interval adjusted for pairing; the same comparison per domain is exploratory and uncorrected. The between-system hypothesis (H4) used Cochran's Q on first-run composite concordance in 40 scenario-language blocks, with Holm-adjusted pairwise McNemar tests. Reproducibility (H3), identical codes across the three runs at the scenario, system and language level, is assessable on d1 and d2 only: d1 on all 60 paired cells per language, d2 on the 30 antibiotic-indicated paired cells in which it is applicable; the language contrast used the exact McNemar test on the paired cells. Safety-critical error rates are reported with confidence intervals on the first run. Two registered sensitivity analyses repeated the primary comparison with the modal code across runs (sensitivity 1) and restricted to dose and duration (sensitivity 2). No power calculation was performed: the benchmark size was set by purposive coverage of the clinical situations and by the feasibility of double-coding 360 responses, so estimates carry limited precision and are interpreted with their confidence intervals; system-specific results are descriptive. Tests were two-sided at alpha 0.05. Three post hoc checks, labelled as such, were added after review of the primary result: an exact scenario-level sign-flip permutation test and a scenario-clustered bootstrap (20 scenarios, 10,000 resamples), both addressing the clustering of the three systems within scenarios that the 60-pair test ignores; the primary comparison from each rater's independent first-run codes; and the primary comparison under a stricter range rule and other alternative readings of the clarified scoring rules.

Data availability

The registered protocol, scenarios and key, prompts in both languages and the translation audit trail are deposited with the registration. The deviations log, the 360 normalised responses, the manifests, all rating and re-scoring sheets as returned, the adjudication records, the consensus dataset with a data-integrity statement and verification script, and the analysis script that reproduces every figure in this article are deposited in the OSF project (https://osf.io/rbq3w). The coding manual, run order sheet, guideline verification log and preliminary paid-tier responses are added to the project at submission; the blinding map is released with the final deposit.

## Results

Data set

All 360 registered responses were collected and none was excluded; no response was a refusal, a clarifying question, a non-answer or a language mismatch. Median response length was shorter for Georgian than for English prompts in all three systems (Claude 336 versus 486 words, ChatGPT 372 versus 405, Gemini 236 versus 304), although word counts are not comparable across languages of different morphology.

Inter-rater agreement

First-round agreement on the applicable cells of the 168 shared responses ranged from 82 to 100 per cent by domain, with kappa moderate for agent and dose and substantial for duration (Table 2); kappa is not estimable where both raters used one category. The 85 agent cells include the eight S14 responses in which R1 entered NA. On the re-scoring of domains 2 to 4, agreement was 98.3 per cent for agent (kappa 0.93, 95 per cent CI 0.74 to 1.00), 96.3 for dose (0.89, 0.68 to 1.00) and 90.7 for duration (0.77, 0.55 to 0.94); the eight differing cells were adjudicated, five as R1 had coded them and three as R2 had, three of the seven Georgian cells in favour of concordance. On the re-scoring of repeat-run agent codes, the raters agreed on all 120 cells.

**Table 2:** Agreement between the two independent raters on the applicable cells of the 168 shared responses NE, not estimable (both raters used a single category). NI, not interpreted (R1 showed no variation in this domain).

Variable	Applicable cells	Agreement (%)	Cohen's kappa	Kappa 95% CI lower	Kappa 95% CI upper
d1 decision	168	100	NE	NE	NE
d2 agent	85	85.9	0.36	0.09	0.62
d3 dose	54	81.5	0.44	0.14	0.71
d4 duration	54	90.7	0.77	0.56	0.94
d5 referral	120	98.3	NI	NI	NI
Harmful error	120	100	NE	NE	NE

Primary outcome: concordance by prompt language

On the first run, 57 of 60 English responses (95.0 per cent, 95 per cent CI 86.3 to 98.3) and 46 of 60 Georgian responses (76.7 per cent, 95 per cent CI 64.6 to 85.6) were fully concordant with the reference key. Of the 60 scenario-system pairs, 47 were concordant in both languages, 12 were concordant in English only and one in Georgian only. The paired difference was 18.3 percentage points in favour of English (95 per cent CI 7.5 to 29.2; exact McNemar p = 0.003). The primary hypothesis was supported.

The difference was confined to the scenarios in which an antibiotic is indicated. All 30 pairs from the ten no-antibiotic scenarios were fully concordant in both languages. Among the 30 pairs from the antibiotic-indicated scenarios, 27 were fully concordant in English and 16 in Georgian (90.0 versus 53.3 per cent; discordant 12 versus 1, exact McNemar p = 0.003). This decomposition of the primary outcome was not registered and is reported as descriptive; it shows that the subset carries the whole of the language difference (Table 3).

**Table 3:** Concordance by domain and prompt language, first run, paired by scenario and system EN, English; KA, Georgian; CI low and CI high, lower and upper bounds of the Wilson 95 per cent confidence interval of the percentage; Diff., difference in percentage points (English minus Georgian); p, exact McNemar test. Domain-level tests are exploratory and not corrected for multiplicity. Pairs in which the domain is not applicable to the scenario are excluded.

Domain	Pairs	EN n	EN %	EN CI low	EN CI high	KA n	KA %	KA CI low	KA CI high	Difference (points)	McNemar p
d1 decision	60	60	100	94	100	60	100	94	100	0	1
d2 agent	30	30	100	88.6	100	21	70	52.1	83.3	30	0.004
d3 dose	27	26	96.3	81.7	99.3	16	59.3	40.7	75.5	37	0.006
d4 duration	27	25	92.6	76.6	97.9	15	55.6	37.3	72.4	37	0.002
d5 referral	60	59	98.3	91.1	99.7	59	98.3	91.1	99.7	0	1
Composite	60	57	95	86.3	98.3	46	76.7	64.6	85.6	18.3	0.003
Composite, antibiotic-indicated scenarios (post hoc decomposition)	30	27	90	74.4	96.5	16	53.3	36.1	69.8	36.7	0.003

By domain (Table 3, Figure 1A; the test of H2), the decision was concordant in every one of the 360 responses and referral advice in 59 of 60 per language. Of the 60 first-run responses to the 10 no-antibiotic scenarios, 48 (26 English, 22 Georgian) advised against starting an antibiotic but offered a back-up prescription should the condition worsen; these were scored as concordant decisions because no antibiotic was recommended at the visit. Under a stricter reading, in which any prescription offered is a departure from "no antibiotic", decision concordance in those scenarios would be 12 of 60, equally in both languages. The 100 per cent figure therefore describes the decision to start an antibiotic, not the absence of any antibiotic advice. The difference arose in the regimen domains: an accepted first-choice agent was named in all 30 assessable English responses and 21 of 30 Georgian (p = 0.004); dose was concordant in 26 of 27 English and 16 of 27 Georgian responses (p = 0.006) and duration in 25 of 27 and 15 of 27 (p = 0.002). Dose and duration are hierarchical under rule A2b: when the agent was discordant they were coded 0, so their margins partly inherit the agent result. Among responses with an accepted agent, dose was discordant in one English and two Georgian responses and duration in two English and three Georgian, the latter all five- to seven-day courses where the key specifies three days; these counts are descriptive. The 17 responses that were not fully concordant are listed in Table 4. All nine Georgian agent discordances were a broader or combination first-choice regimen: co-amoxiclav in eight, amoxicillin plus metronidazole in one. The one pair concordant in Georgian only was a paediatric scenario in which the English response gave a per-dose amount below the accepted band. Three post hoc checks supported the direction of the result. The scenario-level sign-flip test, a conservative robustness analysis that treats each scenario as one unit, gave p = 0.016 (11 more concordant English than Georgian responses over 20 scenarios; 12 scenarios contributed no difference), and the scenario-clustered bootstrap gave a 95 per cent confidence interval of 6.7 to 30.0 points for the paired difference; the headline p of 0.003 rests on the registered 60-pair analysis and the scenario-level p of 0.016 is the more conservative figure. From each rater's independent first-round codes, before adjudication and re-scoring, the comparison was 56 of 60 versus 45 of 60 for R1 (p = 0.001) and 55 of 60 versus 45 of 60 for R2 (p = 0.006). Under a stricter range rule, one English response became discordant and the comparison was 56 of 60 versus 46 of 60 (p = 0.006).

**Figure 1:**
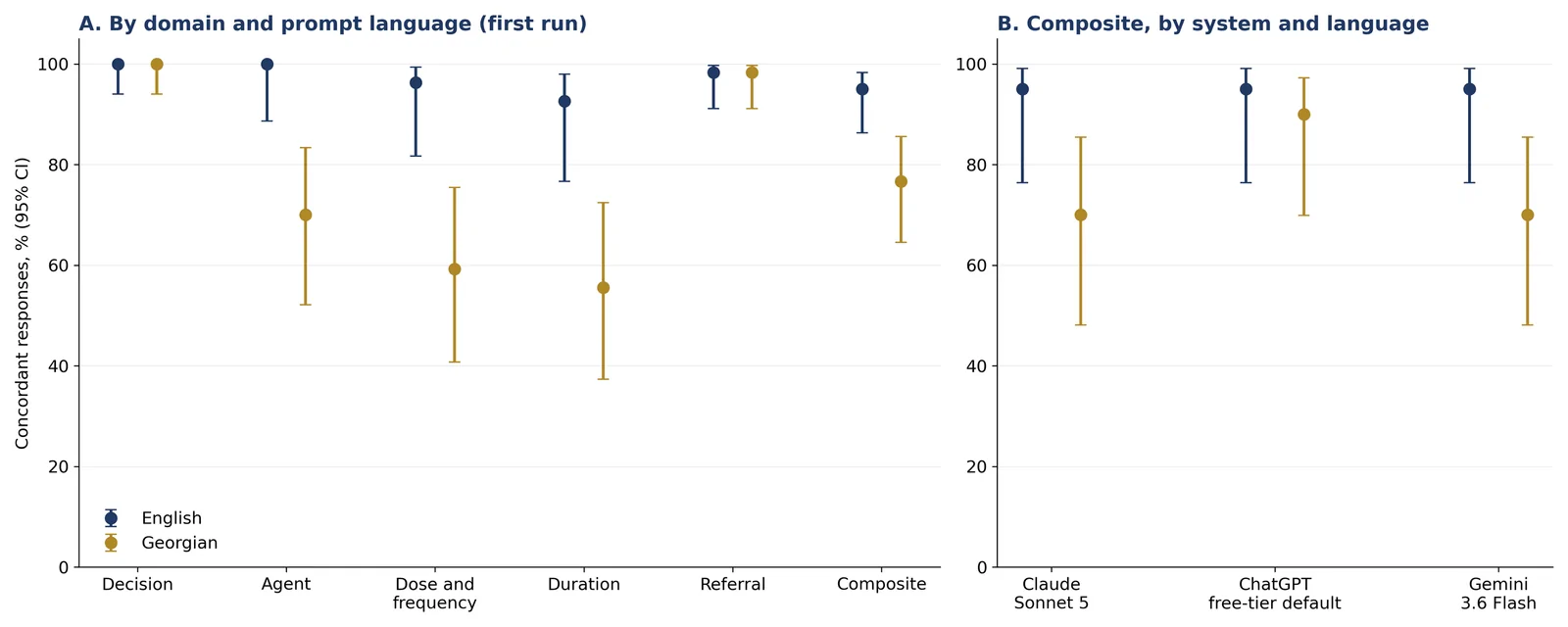
Guideline concordance by prompt language A: proportion of first-run responses concordant with the reference key in each domain and on the composite, paired by scenario and system; the agent domain is assessable in 30 pairs and dose and duration in 27. B: composite concordance for each system, 20 responses per language. Points are proportions with Wilson 95 per cent confidence intervals.

**Table 4:** The 17 first-run responses that were not fully concordant, with the regimen each presented as first choice and the reference standard TDS, three times daily; QDS, four times daily; BD, twice daily. Doses are as stated in the response; where the response named alternatives, only the regimen presented as first choice is shown. Under addendum rule A2b, dose and duration are not assessed when the first-choice agent is discordant.

Language	Scenario	System	Discordant domain	Second discordant domain	First-choice regimen as stated	Reference (key v7.2)
English	S13 pericoronitis, systemic	Claude	Duration (not stated)		Amoxicillin 500 mg TDS, with or without metronidazole 400 mg TDS, as a bridge before admission; duration deferred to the admitting team	Metronidazole 400 mg TDS or amoxicillin 500 mg TDS, 3 days
English	S13 pericoronitis, systemic	Gemini	Duration	referral	Amoxicillin 500 mg TDS, 5 days; no urgent-referral criteria stated	Metronidazole 400 mg TDS or amoxicillin 500 mg TDS, 3 days; urgent referral criteria
English	S20 severe infection, child 14 kg	ChatGPT	Dose		Amoxicillin 225 mg TDS, 5 days	Amoxicillin 250 mg TDS (up to 30 mg/kg TDS), 3 to 5 days
Georgian	S11 spreading infection	Claude	Agent (combination)		Amoxicillin 500 mg plus metronidazole 400 mg TDS, 5 to 7 days	Phenoxymethylpenicillin 500 mg QDS or amoxicillin 500 mg TDS, 3 to 5 days
Georgian	S11 spreading infection	Gemini	Agent		Co-amoxiclav 875/125 mg BD, 5 to 7 days	As above
Georgian	S13 pericoronitis, systemic	ChatGPT	Agent		Co-amoxiclav 875/125 mg BD, 5 days	Metronidazole 400 mg TDS or amoxicillin 500 mg TDS, 3 days
Georgian	S13 pericoronitis, systemic	Claude	Duration		Amoxicillin 500 mg plus metronidazole 400 mg TDS, 5 to 7 days	As above
Georgian	S13 pericoronitis, systemic	Gemini	Agent	referral	Co-amoxiclav 875/125 mg BD, 5 to 7 days; no urgent-referral criteria stated	As above; urgent referral criteria
Georgian	S15 impaired host defence	Claude	Agent		Co-amoxiclav 500/125 mg TDS, 5 to 7 days	Phenoxymethylpenicillin 500 mg QDS or amoxicillin 500 mg TDS, 3 to 5 days
Georgian	S15 impaired host defence	Gemini	Agent		Co-amoxiclav 875/125 mg BD, 5 to 7 days	As above
Georgian	S16 penicillin anaphylaxis	ChatGPT	Dose		Azithromycin 500 mg once daily, 3 days, extendable to 5	Azithromycin 500 mg on day 1 then 250 mg daily, 5 days in total (or metronidazole, clarithromycin, clindamycin)
Georgian	S17 necrotising gingivitis	Claude	Duration		Metronidazole 400 mg TDS, 5 to 7 days	Metronidazole 400 mg TDS or amoxicillin 500 mg TDS, 3 days
Georgian	S17 necrotising gingivitis	Gemini	Duration		Metronidazole 250 to 500 mg TDS, 5 to 7 days	As above
Georgian	S18 post-extraction infection	Claude	Agent		Co-amoxiclav 625 mg TDS, 5 to 7 days	Phenoxymethylpenicillin 500 mg QDS or amoxicillin 500 mg TDS, 3 to 5 days
Georgian	S18 post-extraction infection	Gemini	Agent		Co-amoxiclav 875/125 mg BD, 5 to 7 days	As above
Georgian	S20 severe infection, child 14 kg	Claude	Dose		Amoxicillin 200 mg TDS, 5 to 7 days	Amoxicillin 250 mg TDS (up to 30 mg/kg TDS), 3 to 5 days
Georgian	S20 severe infection, child 14 kg	Gemini	Agent		Co-amoxiclav suspension 40 to 45 mg/kg/day in two doses, 5 to 7 days	As above

Concordance by system

With both languages pooled, first-run composite concordance was 37 of 40 for ChatGPT (92.5 per cent, 95 per cent CI 80.1 to 97.4) and 33 of 40 for both Gemini and Claude (82.5 per cent, 68.0 to 91.3); Cochran's Q was 4.00 (p = 0.14) and no pairwise difference was significant. All three systems were concordant in 19 of 20 English responses; Georgian counts were 18 of 20 for ChatGPT and 14 of 20 for Gemini and for Claude (Figure 1B). No system-by-language interaction was registered, and these stratified counts are descriptive.

Reproducibility across runs

Reproducibility was assessable on the decision and agent domains only. The decision code was identical across the three runs in all 120 cells. The agent domain is not applicable in the 30 no-antibiotic cells per language, so the informative denominator is the 30 antibiotic-indicated cells, where the agent code was identical across runs in 29 of 30 English (96.7 per cent, 95 per cent CI 83.3 to 99.4) and 26 of 30 Georgian cells (86.7 per cent, 70.3 to 94.7); paired, three cells were reproducible in English only and none in Georgian only (p = 0.25). The hypothesis of lower reproducibility in Georgian was not supported on the domains where it could be tested. In each non-reproducible cell the first-choice agent switched between an accepted agent and co-amoxiclav or a combination across runs. An earlier figure of 60 of 60 versus 54 of 60 (p = 0.031), obtained before the repeat-run codes were re-scored under the first-choice rule, compared two scoring rules and is withdrawn (D22).

Harmful errors

No first-run response met any criterion on the closed list of safety-critical errors: 0 of 60 per language (95 per cent CI 0.0 to 6.0) and 0 of 40 per system (0.0 to 8.8). Repeat runs were not coded for this variable. These are upper bounds for these scenarios, systems and dates; the interval is compatible with a true rate of up to 6 per cent, and the closed list does not cover every clinically important error.

Sensitivity analyses

Sensitivity analysis 1 could not be performed as registered, because a modal composite cannot be formed when repeat runs are coded on d1 and d2 only. A restricted version on those two domains, not equivalent to the registered analysis, gave concordance under the modal code in 29 of 30 English and 23 of 30 Georgian antibiotic-indicated cells, where the difference lies (paired difference 10.0 points over all 60 cells, 95 per cent CI 2.4 to 17.6; p = 0.031; the 30 no-antibiotic cells are concordant by construction).

Sensitivity analysis 2, restricted to dose and duration, gave concordance on both in 24 of 27 English pairs (88.9 per cent, 95 per cent CI 71.9 to 96.1) and 13 of 27 Georgian pairs (48.1 per cent, 30.7 to 66.0; p = 0.003); under rule A2b this is not independent of agent selection. Because the scoring rules were clarified after the first coding round, the primary comparison was also recomputed under each alternative reading (Table 5). Under every reading that scores the regimen presented as first choice, the difference lies between 16.7 and 21.7 points and remains significant; under the most permissive reading possible, which credits the regimen domains of the nine Georgian responses with a non-accepted first-choice agent as concordant, it falls to 5.0 points and is not significant. The language-associated difference is therefore robust if the regimen a response presents as its primary recommendation is the unit of scoring, the interpretation specified in the finalised coding addendum; it is not robust to every conceivable rule.

**Table 5:** Primary comparison under alternative readings of the scoring rules clarified after the first coding round (post hoc) All readings are post hoc; A is the analysis reported in the Results. A: first-choice agent scored; a stated range is concordant if it contains or overlaps the accepted value; dose and duration not assessed under a discordant agent; combination regimens scored as a whole. B: a stated range must lie entirely within the accepted value. C: overlap of a stated range with an accepted range is not credited. D: a combination regimen is scored on its accepted component. E: B and C applied together. F: the regimen domains of the nine Georgian responses with a discordant first-choice agent set to concordant; decision and referral unchanged.

Reading	English, n/60	Georgian, n/60	Difference (points)	Pairs concordant in English only	Pairs concordant in Georgian only	McNemar p
A. Final rules, as reported	57	46	18.3	12	1	0.003
B. Stricter range rule	56	46	16.7	11	1	0.006
C. Range overlap not credited	57	44	21.7	13	0	<0.001
D. Combination scored on its accepted component	57	47	16.7	11	1	0.006
E. Rules B and C combined	56	44	20	12	0	<0.001
F. Permissive agent rule, upper bound	57	54	5	4	1	0.375

Descriptive variables

Descriptive variables, coded by one rater on the first run, are reported without inference. A delayed prescription was suggested in 26 of 60 English and 22 of 60 Georgian responses, a source was cited in 28 and 24, and the response deferred to local guidance in 27 and 21.

## Discussion

In this preregistered evaluation of three consumer LLM interfaces on 20 purposively selected benchmark scenarios, matched prompts were fully concordant with the reference key in 57 of 60 English and 46 of 60 Georgian first-run responses, a paired difference of 18.3 points, with 12 pairs concordant in English only and one in Georgian only. The inference is conditional on these scenarios and these three products, not an estimate of a language effect across dentistry. The difference was not in the decision to prescribe, which was concordant in all 360 responses, nor in referral advice. The regimen difference least affected by the hierarchical scoring rule was agent selection: 30 of 30 accepted first-choice agents in English against 21 of 30 in Georgian. Dose and duration were also lower in Georgian but inherited agent errors under rule A2b; where the agent was accepted, the remaining discordances were few in both languages. No response met a prespecified safety-critical error criterion, within an upper bound of 6 per cent per language. The direction of the difference was present in each rater's independent coding, remained significant with scenarios as the unit (p = 0.016), and had a scenario-clustered interval excluding zero. Reproducibility of the agent code did not differ significantly between languages (29 of 30 versus 26 of 30 antibiotic-indicated cells, p = 0.25), a result confined to the decision and agent domains.

Two features are notable. The difference appeared only where an antibiotic was indicated: all 30 no-antibiotic pairs were concordant in both languages, the systems declining antibiotics for pulpitis, localised abscess with drainage, dry socket, routine extraction, and the other presentations for which the key specifies none. The three systems were identical in English (19 of 20 each) and separated only in Georgian (18, 14 and 14 of 20); as the pooled test was not significant and no interaction was registered, these stratified counts are descriptive and not evidence that one product is superior in Georgian.

The English-language findings are broadly consistent with previous vignette studies. Nguyen et al. found that six LLMs identified the diagnosis and the decision to prescribe in 88 to 100 per cent of general-practice vignettes, including dental infections, and chose an appropriate agent in most, but gave a guideline-concordant dose and duration in only 0 to 75 per cent and were less reliable when applying country-specific guidance [10]. De Vito et al. found substantial between-system variation in dosing and duration across 60 infection cases [11]. In dentistry, Çırakoğlu et al. reported high scores on general questions about antibiotic use with weaker performance on patient-specific detail [7], and Arslanparcasi and Arslanparcasi found that four conversational systems generally identified antibiotic indications in endodontic scenarios but with frequent incomplete answers [8]. Across these studies, broad treatment decisions tend to be more reliable than complete, guideline-specific regimens. Our data extend that pattern by showing lower full guideline concordance for Georgian than for matched English prompts, while decision concordance remained unchanged in this benchmark.

The language result also extends prior cross-lingual work. Jin et al. documented lower correctness, consistency and verifiability for health queries in Spanish, Chinese and Hindi than in English [12]; multilingual medical benchmarks have likewise shown performance variation across languages [13], including German, French and Italian medical questions [14]. Georgian is a low-resource Kartvelian language with a distinct script, and recent benchmarking has only begun to characterise LLM reasoning performance in Georgian [15]. To our knowledge, this is the first evaluation of guideline-concordant clinical antibiotic prescribing advice in Georgian. In this benchmark, decision and referral components were preserved across languages, while the guideline-specific regimen components showed lower concordance in Georgian.

Several mechanisms could produce this pattern; none was measured and all remain hypotheses. English is disproportionately represented in medical language-model resources [13] and tokenisation efficiency differs widely across languages [19], but training corpora, tokenisers and token counts were not examined. The error pattern itself points to agent selection: eight of nine Georgian agent discordances were co-amoxiclav as first choice and the ninth a combination. The shorter Georgian responses did not simply omit the regimen: the code for an agent named without a dose or duration was used once, for an English response, so the Georgian discordances were regimens stated in full that fell outside the accepted values, and they departed from the reference in one direction, towards a broader agent and, where duration was assessable independently, a longer course. Subtle translation effects remain possible despite human translation, back-translation and pre-testing. The tendency towards broader first-line therapy may reflect Georgian-language training material or regional prescribing practice, neither of which was measured. The study establishes a language-associated difference in this benchmark, not its mechanism.

Between-system differences should be interpreted cautiously. The pooled comparison was not significant (p = 0.14), no pairwise contrast was significant after Holm adjustment, and the study was not powered for product-to-product comparisons. The systems were evaluated as their free-tier consumer defaults on two days in September 2026. Gemini responses were generated with web search available because the interface did not permit it to be disabled, so for one of the three systems the language contrast may include a language-dependent effect of browsing, and the ChatGPT model version was not displayed and could only be recorded from model self-report. These choices were intended to approximate ordinary free-tier use, but they also mean that the system-level findings describe specific consumer products, as served to a single free account on two days, rather than stable underlying models; account-level or regional routing and product experiments were not sampled.

The clinical implication is narrow. In these 20 constructed vignettes, the systems consistently reached the reference-concordant decision and nearly always gave concordant referral advice, but were less likely to give a fully concordant regimen in Georgian. Explicit vignette features may have made the decision unusually easy, and the safety-critical error interval is wide, so the findings do not support delegating prescribing decisions to an LLM; they support checking agent, dose, frequency and duration against an authoritative reference, particularly for recommendations generated in Georgian. Asking in English may improve concordance in this benchmark but is no substitute for reliable Georgian-language guidance.

For policy, the findings underscore the value of accessible, authoritative Georgian-language dental prescribing guidance, for which a national guideline is the obvious vehicle: first as a local reference against which any recommendation can be checked. Whether it would also improve what consumer models return in Georgian is not shown here, since publication does not ensure uptake into proprietary training data, although it could support retrieval-based tools, evaluation and local adaptation. The absence of safety-critical errors is reassuring within this benchmark but is not a safety guarantee.

The study was preregistered with a scenario-level reference key and a public audit trail of every amendment, used human translation with independent back-translation, blinded responses to system and run, used two independent dentist raters, and documented adjudication and every deviation. The paired design controls for scenario and system within the language comparison but not for the order and timing limitations below.

Limitations

Several limitations follow from the deviations and the design. The registered randomised run order was not followed, so calendar position is confounded with scenario, and within each scenario English always preceded Georgian, so language and order are confounded; the primary result is reported as a difference associated with prompt language, not a causal effect of language. Each prompt was sent in a fresh session with memory and personalisation disabled, so context was not carried between prompts, but the server-side state of consumer products, including any caching, cannot be inspected by an external user. The registered modal-composite sensitivity analysis could not be performed, and reproducibility is assessable only on the decision and agent domains, not where the language difference lies. Six repeat-run S14 agent codes entered as NA by one rater were set to concordant by the investigator rather than by a second rater.

The raters could not be blinded to prompt language, so any tendency to score Georgian text more or less strictly acts directly on the primary comparison. The raters who coded the registered dataset had not taken part in the calibration rounds that shaped the manual, which may have contributed to the moderate first-round kappa for agent and dose; the direction and significance of the language difference were nevertheless present in each rater's independent codes. All 29 first-round disagreements and the eight re-scoring disagreements were decided by the principal investigator, blind to rater identity, system and run but not to language; the first-round decisions favoured R2 in 24 of 29, including all eight English disagreements, and three of the seven Georgian re-scoring cells were decided in favour of concordance. The scoring rules that produced the final figures were therefore not fully prespecified: the addendum was written after the first coding round, and the three clarifications the re-scoring adjudication required were written with the affected responses in view: the overlapping-range rule moved three Georgian duration cells to concordant, and the combination-regimen rule moved one Georgian response to discordant. Normalisation removed formatting and self-identification, but stylistic cues may have remained recognisable. Both raters and the adjudicator are authors, and the descriptive variables rest on one rater.

The reference standard is external (SDCEP, with ADA 2019 and AHA 2021 where applicable) because we could identify no Georgian one; a clinician applying a different legitimate standard would score some regimens differently, and two scenarios sit where sources diverge. The key for S13, pericoronitis with systemic involvement, expects an oral regimen with urgent-referral advice, because the vignette describes fever, trismus and difficulty with solid food but not the airway compromise that defines S14, where only the decision and emergency transfer are scored; a reader who judges that S13 already warrants admission would score its regimen differently, and one discordance per language arises there. The composite is structurally easier to achieve in no-antibiotic scenarios, where only decision and referral are scored, so with the balanced 10:10 case mix it describes benchmark performance, not prevalence-weighted accuracy. Concordance with this reference is not the same as clinical inappropriateness: a seven-day amoxicillin course is discordant with the SDCEP key but within the ADA regimen for conditions in its scope [3]; the safety-critical error list, not the composite, measures dangerous advice, and that list does not cover every important error. Under rule A2b, the dose and duration margins inherit a discordant agent and are not stand-alone error rates. The choice of a UK standard therefore shapes the result more than a single sentence conveys: co-amoxiclav as first choice and five to seven-day courses are common practice in many countries, and Table 5 (reading F) bounds their contribution, since crediting them reduces the language difference to 5.0 points (p = 0.375). What the standard cannot explain is the asymmetry: the English responses to the same scenarios, from the same systems, did not make the same departures.

The key was amended twice and domains 2 to 4 were re-scored after two interpretive rules were found to have been applied inconsistently. The primary estimate moved across these stages: 16.7 points in the first coding round, 8.3 points in an intermediate pass before the first-choice rule was applied uniformly, 20.0 points when one re-scoring sheet was temporarily treated as consensus, and 18.3 points after both original sheets were compared and the eight differing cells adjudicated; the full sequence is in the public deviations log. The final analysis applies the first-choice rule uniformly to all three runs and the independent-rater and scenario-level checks support the same direction, but the magnitude is sensitive to how multiple-option recommendations are scored: Table 5 shows 16.7 to 21.7 points under every reading that scores the regimen presented as the primary recommendation, and 5.0 points, not significant, under the most permissive reading. The result should be read as robust conditional on that unit of scoring.

Twenty scenarios and 60 pairs give limited precision for domain- and system-level comparisons, which were not the basis of a power calculation. Prompts were single-turn, fixed-format vignettes with explicit clinical features, so complete decision concordance may reflect a ceiling effect and may not generalise to ambiguous real encounters. Each system was sampled through one free account on two days, so account-level or regional routing was not sampled, and consumer systems change without notice [20]; the results describe the free-tier defaults of September 2026.

Three extensions follow. Coding dose, duration and referral on the repeat runs would make reproducibility assessable where the language difference lies and would restore the registered modal-code sensitivity analysis. Repeating the protocol at intervals with the same frozen prompts and key would show whether the gap changes as products change. Supplying a concise authoritative guideline in the prompt would show whether access to a reference narrows the gap, without presuming the mechanism.

## Conclusions

When matched dental scenarios were put to three consumer LLM interfaces in Georgian and English, the guideline-concordant decision on whether to prescribe was preserved across languages, while full concordance with a UK stewardship standard was lower for Georgian prompts. The most directly interpretable difference was first-choice agent selection: Georgian responses more often recommended broader-spectrum or combination therapy than the reference accepts. No response met a prespecified safety-critical error criterion, although the interval does not exclude a non-zero rate and the closed list does not cover a broader agent or a longer course. The findings support verification of LLM-generated regimen details against an authoritative source and underscore the value of accessible, authoritative Georgian-language prescribing guidance; they establish neither the safety of LLM prescribing nor the mechanism of the difference.
